# The Association Between Pregnancy-Related Factors and Health Status Before and After Childbirth With Satisfaction With Skilled Delivery in Multiple Dimensions Among Postpartum Mothers in the Akatsi South District, Ghana

**DOI:** 10.3389/fpubh.2021.779404

**Published:** 2022-02-01

**Authors:** Lawrence Sena Tuglo, Comfort Agbadja, Cynthia Sekyere Bruku, Vivian Kumordzi, Jessica Dzigbordi Tuglo, Leticia Atiah Asaaba, Mercy Agyei, Cynthia Boakye, Sylvia Mawusinu Sakre, Qingyun Lu

**Affiliations:** ^1^Department of Epidemiology, School of Public Health, Nantong University, Nantong, China; ^2^Department of Nutrition and Dietetics, School of Allied Health Sciences, University of Health and Allied Sciences, Ho, Ghana; ^3^Diettherapy Department, Ho Teaching Hospital, Ho, Ghana; ^4^Department of Midwifery, School of Nursing and Midwifery, University of Health and Allied Sciences, Ho, Ghana; ^5^Obstetrics and Gynaecology Department, Saint (ST) Dominic Hospital, Akwatia, Ghana; ^6^Ashaiman Municipal Health Directorate, Ashaiman, Ghana; ^7^Community Health Department, Evangelical Presbyterian Mimi Clinic, Adaklu, Ghana; ^8^Maternity Department, Madina Polyclinic Kekele, Madina, Ghana; ^9^Maternity Department, Ga South Municipal Hospital, Waija, Ghana; ^10^Maternity Department, Eastern Regional Hospital, Koforidua, Ghana; ^11^Department of Community Health Nursing, Nurses Training College, Ho, Ghana

**Keywords:** pregnancy-related factors, health status before and after childbirth, satisfaction, skilled delivery, multiple dimensions, postpartum mothers, Ghana

## Abstract

**Background:**

Skilled delivery has been a pronounced concern and has been investigated over the years in developing countries. An inclusive understanding of the satisfaction of postpartum mothers is vital in improving the quality of skilled delivery, which is beneath the standard in some parts of developing countries. This study assessed the association between pregnancy-related factors and health status before and after childbirth with satisfaction with skilled delivery in multiple dimensions among postpartum mothers in the Akatsi South District, Ghana.

**Methods:**

A community-based, cross-sectional study was conducted among 538 postpartum mothers who participated through the systematic sampling method. Data collection was performed through a pretested and structured questionnaire developed from the WHO responsiveness concept and other prior studies. Questions on satisfaction were categorized into six dimensions. The associations were determined using bivariable and multivariable logistic regression analyses.

**Results:**

The overall satisfaction of postpartum mothers with skilled delivery was 80.7%. The highest (89.6%) and the lowest (12.8%) satisfaction with skilled delivery were found in technical quality and financial dimensions. Analysis revealed that autonomously age and delivery procedure were significantly associated with the dimensions of communication and responsiveness. Postpartum mothers who delivered at private healthcare facilities [crude odds ratio (COR) = 1.70; (95% CI 1.00–2.90); *p* = 0.049] had preterm pregnancy before delivery [COR = 2.08; (95% CI 1.02–4.21); *p* = 0.043], had cesarean section [COR = 2.73; (95% CI 1.05–7.12); *p* = 0.040], and presented with complications after childbirth [COR = 2.63; (95% CI 1.09–6.35); *p* = 0.032] were more likely to be satisfied in the dimension of communication only compared to their counterparts. Regarding responsiveness, multiparous mothers [COR = 1.63; (95% CI 1.06–2.51); *p* = 0.007] were more likely to be satisfied than primiparous mothers. Overall satisfaction was significantly and positively correlated with the various dimensions of skilled delivery.

**Conclusions:**

The majority were satisfied with five dimensions of satisfaction with skilled delivery except for the financial dimension. The District Health Directorate of Akatsi South should take into consideration these findings in their policy development for forward-looking skilled delivery.

## Key Messages

### What Is Already Known on the Subject?

Several studies have found an association between demographics and satisfaction with skilled delivery. The majority of these studies conducted in Ghana have assessed satisfaction in general. No study was found to assess how pregnancy-related factors and health status before and after childbirth might affect satisfaction with skilled delivery.

### What Does This Study Add?

Our study assessed satisfaction with skilled delivery in multiple dimensions and found the highest satisfaction in technical quality and lowest satisfaction in the financial dimension. This study found that parity, pregnancy status, mode of delivery, delivery procedure, and presence of complications were associated with satisfaction with skilled delivery, specifically in the dimensions of communication and responsiveness.

## Introduction

The WHO has estimated that 295,000 maternal deaths occur globally from conception to childbirth and during the postpartum period (1, 2). The majority are from developing countries where there is inadequate access to skilled delivery (2–4). The provision of delivery services by skilled health personnel is essential to improving maternal health (4, 5). Over the years, the emphasis on maternal care in developing countries has been extended from the outmoded objective of reducing mortality and morbidity to wider goals, such as improving satisfaction (4, 6). This has earned acceptance to determine the standard of skilled delivery, reproductive care (2–4), and clinical practices. It helps healthcare management to develop policies to meet expectations in the course of improving healthcare delivery (7, 8). This is by the WHO's definition of health as “not just the absence of illness or disease but a state of absolute physical, psychological, and general health”(6). Thus, the WHO basis for quality healthcare services has been linked to the dimensions of providing skilled delivery with satisfaction (2, 9).

The satisfaction of postpartum mothers is the measure of their desired expectations, intentions, preferences, and experiences from skilled delivery (7, 8, 10, 11). Skilled delivery is a service provided in a structured facility by a certified health worker who has been trained to be proficient in the services required to improve the lives of expectant mothers during labor and the postpartum period (2, 12). Approximately 69–73% of maternal and neonatal mortalities could have been prevented among mothers if skilled delivery was made available and accompanied by satisfaction (2). The satisfaction of postpartum mothers has been presumed to be the preeminent adjudicator for the improvement of skilled delivery (5). Understanding the factors associated with the satisfaction of postpartum mothers is essential to provide standard skilled delivery in healthcare facilities (8, 13). The services provided in health facilities at the maternity department are essential to improving maternal and neonatal health to prevent mortality and morbidity due to complications from conception to childbirth (2, 12, 14).

**Figure 1:**
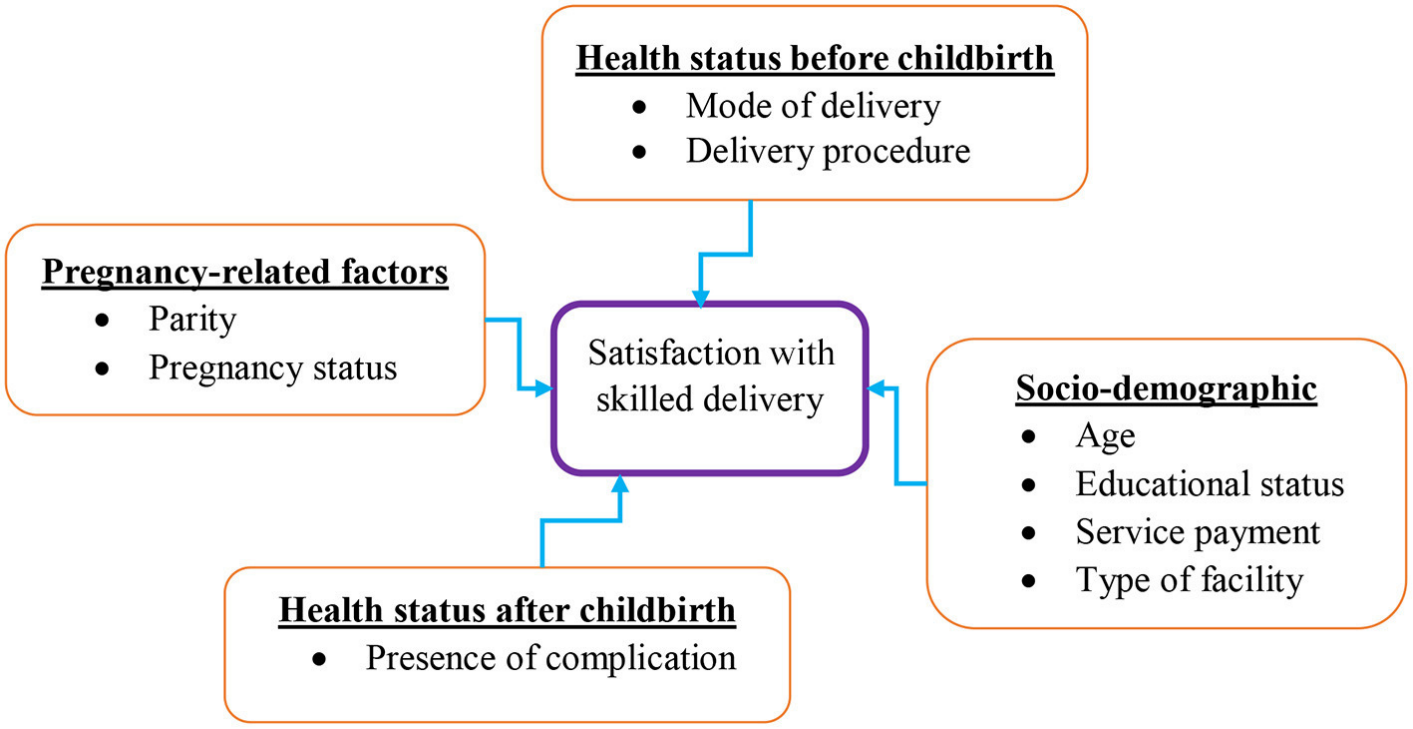
A conceptual framework showing factors associated with satisfaction with skilled delivery.

Studies have found inadequate health professionals, inappropriate prognoses and therapeutics, inadequate needed capital, poor referral systems, preference of skilled personnel, and previous exposure to complications as health system barriers that affect skilled delivery to mothers (2, 5, 12, 15–17). Satisfaction of patients with skilled delivery has been reported to be associated with sociodemographic factors (Figure 1) (1, 4, 7–9, 14, 18, 19), long waiting time (4, 14, 19), treatment outcome (2, 18–20), availability, and preference of healthcare providers (2, 4). Having good health status is essential, particularly for mothers before and after childbirth for several reasons, i.e., the health of the mother and the well-being development of the child (6). Studies have shown that postpartum mothers undergo several psychiatric disorders, such as stigmatization, emotional, and psychological distress during pregnancy and childbirth (6, 9, 21).

The experience of having psychiatric illness has been a reported cause of emotional disorders from pregnancy to the postpartum period that affect satisfaction (22). In addition, the mother's health status before and after childbirth may be affected by factors related to pregnancy, infant health, and non-medical factors, such as sociodemographic characteristics (6, 9, 12, 21, 22). Many studies have assessed patient satisfaction as a general outcome (4, 5, 9, 18, 19, 23, 24), and others have assessed various dimensions with reported different satisfaction levels (general satisfaction, technical quality, communication, financial, responsiveness, and accessibility) (1, 3, 7, 10–12, 14, 16, 21, 25). The assessment of satisfaction in multiple dimensions and understanding the factors associated with women's pregnancy-related factors and health status before and after childbirth are significantly needed to develop appropriate health interventions (Figure 1) (21, 22). However, associations between these factors, such as parity, pregnancy status, mode of delivery, delivery procedure, and presence of complications with the satisfaction of skilled delivery, have not yet been adequately investigated.

In Ghana, an estimated 63–71% of postpartum mothers received skilled deliveries, likened to 80–95% who accessed antenatal care (ANC) from healthcare professionals (4) compared to 45% in Ethiopia (2). The overall service satisfaction of patients with healthcare delivery in the Volta was 61.7%; however, it was lower than the 83.0% in the Western Region and higher than the 55.0% reported in the Ashanti Region (26). Presently, there has been a swift decline in access to skilled delivery among postpartum mothers in the Akatsi South District based on annual health reports (27). This has been attributed to the following: (1) service dissatisfaction about the noncompliance of healthcare professionals with skilled delivery guidelines approved by their regulatory agencies, (2) previous experiences resulting in non-patronage of postnatal care (PNC), (3) perceptions about ANC, and (4) high utilization of home care after delivery. These problems have been presumed to be the cause of the low utilization of skilled delivery (46%), leading to increased maternal and neonatal mortality in the Akatsi South District (27).

The outcomes of this study would be used to improve attendance at healthcare facilities and address the issues regarding dissatisfaction. This would help the District Health Directorate of Akatsi South develop strategies that could increase the utilization of skilled delivery and significantly reduce maternal and neonatal mortality. It would also be supportive for healthcare regulators, administrators, scholars, and researchers to comprehend the degree to which they can plan different approaches to advance the satisfaction of postpartum mothers, thereby improving the quality of skilled delivery. Hence, our study assessed the association between pregnancy-related factors and health status before and after childbirth with satisfaction with skilled delivery in multiple dimensions among postpartum mothers in the Akatsi South District, Ghana.

## Materials and Methods

### Study Setting

This research was conducted in the Akatsi South District of the Volta Region, Ghana. The district is surrounded to the south by the Keta Municipal, to the east by the Ketu North Municipal, to the north by North Tongu District, and to the west by Agotime Ziope District and the Republic of Togo (28). The population of the district is 98,684, representing 46.1% men and 53.9% women (28). The total number of households is 25,758, of which 8,627 are in urban areas and 17,131 are in rural areas (28). Trading and farming are the primary economic activities in the district.

### Study Design

A community-based descriptive cross-sectional study was conducted in the Akatsi South District, Ghana. This study design defined an occurrence that happens visibly, aided in the broad, precise assembly of evidence, and data about the present study.

### Study Population

The study population included postpartum mothers in the urban communities of Akatsi South District, Ghana.

### Eligibility Criteria

The postpartum mothers in the urban communities of Akatsi South District consented to take part in the study. Postpartum mothers who were extremely sick, those who dissented to partake in this study, and those who did not utilize skilled delivery were excluded. Additionally, postpartum mothers in the rural communities were excluded because of the time frame of the study and other factors that might affect their utilization of skilled delivery, such as bad roads, lack of healthcare centers, and inadequate healthcare professionals.

### Sample Size

Yamane's formula *n* = *N*/1+*N*(*e*^2^) (29) was used to determine the sample size of a known study population, where *n* = sample size, *N* = population size, and *e* = margin of error (5%). The total number of households with postpartum mothers in the urban communities of Akatsi South District is 1,672 (28). A non-response rate of 10% was added. This gave an estimated sample size of 355.

### Sampling Technique and Procedure

Data collection was performed through a systematic sampling technique. A precise description of the sample was derived from the study population. This reduced the concerns of bias throughout the data collection by considering only households with postpartum mothers. We used the theory of systematic sampling such that we divided the total households of postpartum mothers with the estimated sample size (1,672/355) to obtain the approximated number (4.709) ≈ 5. Every fifth household in the community with a postpartum mother was contacted and requested to partake in the study from postpartum mother one to postpartum mother five. The dialogue commenced with the fifth postpartum mother in the urban communities. This is persistent by contacting every fifth postpartum mother found and continuing after the completion of the questionnaire. A total of 751 questionnaires were administered and returned together with incomplete questionnaires. However, 538 (71.6%) were fully answered and used for the analysis.

### Data Collection Tool

A structured questionnaire was designed based on prior studies (7–10, 12, 23). The questions on satisfaction with skilled delivery were adapted and standardized according to the Patient Satisfaction Questionnaire (PSQ-III and PSQ-18) developed by RAND Corporation (Santa Monica, CA, USA) (30), the WHO responsiveness concept modified by (21), the Patient Satisfaction with Nursing Care Quality Questionnaire (PSNCQQ) developed by (9), the Self-reporting Questionnaire on Patient Satisfaction (SQPS) developed by (23), the Donabedian quality assessment framework modified by (24), and the Newcastle Satisfaction with Nursing Scale (NSNS) developed by (18) and used in previous research studies (1, 7–10, 12, 18, 21, 23, 24).

### Assessment of Postnatal Mothers' Satisfaction With Skilled Delivery

The second part comprises 20 questions on the satisfaction of postnatal mothers with skilled delivery. The questions were categorized into six dimensions: general satisfaction, technical quality, communication, financial, responsiveness, and accessibility. Each question has a 5-point Likert scale ranging from (1-very dissatisfied, 2-dissatisfied, 3-not sure, 4-satisfied, and 5-very satisfied) (1, 24). Throughout the analysis, the responses of very satisfied and satisfied were scored 1 point each. The responses of very dissatisfied, dissatisfied, and not sure were scored a 0 point each (24). The response of “not sure” was given a zero score (24). The “not sure” responses had been incorporated to allow easiness of answering by postnatal mothers for opinions deliberated by an unsure or uncertainty. Another reason for “not sure” is because the interview was administered sometimes in the presence of relatives, whereby most postpartum mothers felt timid to voice their discontent about some of the disliked services that had been rendered to them. A cumulative percentage method was employed, which considers only the positive answer (31). The total cumulative percentage is calculated and rounded up to 100%. Cumulative percentages ≥75% were considered “satisfied,” and cumulative percentages <75% were considered “dissatisfied” (1, 14, 24).

### Operational Definitions

*General satisfaction:* This refers to the knowledge, skills, communication, and conduct of healthcare professionals in care delivery toward postnatal mothers. *Technical quality and environment:* This refers to the environment, infrastructure, equipment, and sanitation of the hospital. *Communication and interpersonal manner:* This refers to the connections, attitudes, and links built during skilled delivery between healthcare professionals and postnatal mothers. *Financial aspects:* This refers to the economic background of postnatal mothers in accessing healthcare services. *Responsiveness:* This refers to the willingness of health workers to deliver care to postnatal mothers within the desired time frame. *Accessibility and convenience:* This refers to the availability, suitability, closeness, and user-friendliness of healthcare workers in delivering health services.

### Validity and Reliability

The validation of the questionnaire was performed by four professionals on the topic through an extensive literature review. The questionnaire was initially designed in English, transformed into indigenous languages of the respondents, and finally converted to English to ensure consistency and ease of the question. This was done by proficient translators and the main researchers.

### Data Quality Assurance

The research assistants were thoroughly taken through the questionnaire properly before pretesting was done on 15 postpartum mothers at Ho, Ghana to check their understanding of the questions. The answers gained after pretesting were used to mend the question by ensuring clarity, transparency, and simplicity. The accepted and approved questionnaire was interviewer-administered by seven research assistants. The data gathered were analyzed for accuracy and saved in inaccessible folders on the laptop by the key investigators.

### Data Analysis

The collected data were double-checked physically for their entirety before being captured into the Microsoft Excel 2016 database. IBM SPSS (ver 23.0) was used for statistical analysis. Frequencies and percentages were computed. The chi-square (χ^2^) test and bivariate logistic regression were performed to determine the relationship between satisfaction and the study variables. For the individual satisfaction dimension, first, we included all the study parameters in the model. Variables with *p* < 0.05 in the univariate analyses of each dimension of satisfaction were incorporated into multivariate logistic regression analyses. In the concluding model, *p* < 0.05 was deemed statistically significant.

### Ethical Consideration

Before the start of the study, a research proposal was sent, reviewed, and approved by the Research Ethics Committee (REC) of the University of Health and Allied Sciences (UHAS), Ho. The protocol identification number [UHAS-REC No: A.5 [78] 17–18] was granted. Approvals from the leaders in the communities were granted as well. The research assistants introduced themselves, and consent was granted from each postpartum mother. The aims of the study were explained by the research assistants to the postpartum mothers in their dialects. Knowledgeable approval was granted by the postpartum mother by a thumbprint. No place was allowed for personal documentation for confidentiality purposes. The questionnaires were answered individually to certify privacy.

## Results

### Sociodemographic, Pregnancy-Related Factors, and Health Statuses of the Respondents

A total of 538 postpartum mothers were enrolled as participants of the study. The majority (*n* = 347; 64.5%) were aged between 25 and 40 years. Most n = 283 (52.6%) had attained secondary education. Half (*n* = 270; 50.2%) paid for their services by cash and the National Health Insurance Scheme (NHIS). The majority (*n* = 436; 81.0%) patronized government healthcare facilities. Above three-quarters (*n* = 422; 78.4%) were multipara. Regarding pregnancy status, *n* = 477 (88.7%) had full-term pregnancies before delivery. A high proportion (*n* = 499; 92.8%) had spontaneous vaginal delivery. The majority (*n* = 338; 62.8%) rated the delivery procedures as fair. A high proportion (91.6%), *n* = 493, had no complications after childbirth (Table 1).

**Table 1:** Sociodemographic characteristics, pregnancy-related factors and health statuses of the respondents (*n* = 538).

**Variable**	**Frequency**	**Percentage**
Total	538	100.0
Age (years)		
<25 years	75	13.9
25–40 years	347	64.5
>40 years	116	21.6
Educational status		
None	19	3.5
Basic	103	19.1
Secondary	283	52.6
Tertiary	133	24.7
Service payment		
Cash only	119	22.1
NHIS only	149	27.7
Cash and NHIS	270	50.2
Type of facility		
Private	102	19.0
Government	436	81.0
Parity		
Primipara	116	21.6
Multipara	422	78.4
Pregnancy status		
Preterm	61	11.3
Full-term	477	88.7
Mode of delivery		
Cesarean section	39	7.2
Spontaneous vaginal delivery	499	92.8
Delivery procedure		
Poor	81	15.1
Fair	338	62.8
Good	119	22.1
Presence of complication		
Yes	45	8.4
No	493	91.6

*Data are presented as the frequency and percentage, NHIS-National Health Insurance Scheme*.

### Multiple Dimensions of Satisfaction of Postpartum Mothers With Skilled Delivery

#### Dimension of General Satisfaction

Below half (*n* = 236; 43.9%) of postpartum mothers were satisfied with often receiving counseling during a visit to the facility; *n* = 326 (60.6%) were very satisfied with health workers being interested in providing skilled services; *n* = 224 (41.6%) were very satisfied with midwives giving individual attention to the client; and *n* = 271 (50.4%) were satisfied with instructions provided on medications and follow-up care (Table 2).

**Table 2:** Multiple dimensions of satisfaction of postpartum mothers with skilled delivery (*n* = 538).

**Parameter**	**Responses** ***n*** **(%)**				
	**VD**	**D**	**NS**	**S**	**VS**
**General satisfaction**					
I often receive counseling during a visit to the facility	19 (3.5)	48 (8.9)	21 (3.9)	236 (43.9)	214 (39.8)
Health workers are interested in providing skilled services	19 (3.5)	18 (3.3)	5 (0.9)	170 (31.6)	326 (60.6)
Midwives give individual attention to client	34 (6.3)	22 (4.1)	45 (8.4)	213 (39.6)	224 (41.6)
Instructions on medications and follow-up care	25 (4.6)	40 (7.4)	18 (3.3)	271 (50.4)	184 (34.2)
**Technical quality**					
The facility has everything needed to provide complete medical care	25 (4.6)	23 (4.3)	17 (3.2)	217 (40.3)	256 (47.6)
Competence of health workers	12 (2.2)	21 (3.9)	11 (2.0)	252 (46.8)	242 (45.0)
Professional appearance of health workers	15 (2.8)	13 (2.4)	4 (0.7)	295 (54.8)	211 (39.2)
Good hygienic condition at the hospital	12 (2.2)	8 (1.5)	9 (1.7)	243 (45.2)	266 (49.4)
**Communication**					
I get an explanation of diagnosis, treatment and the reason for medical tests.	14 (2.6)	14 (2.6)	9 (1.7)	256 (47.6)	245 (45.5)
The readiness of the healthcare professional to attend to the client	21 (3.9)	10 (1.9)	5 (0.9)	286 (53.2)	216 (40.1)
I receive care in a very friendly and courteous manner	10 (1.9)	15 (2.8)	8 (1.5)	225 (41.8)	280 (52.0)
**Financial**					
I pay for bills more than I can afford	125 (23.2)	180 (33.5)	42 (7.8)	76 (14.1)	115 (21.4)
I receive the medical service I want without a financial setback	33 (6.1)	50 (9.3)	14 (2.6)	221 (41.1)	220 (40.9)
Items purchase for medical care and use are expensive	151 (28.1)	83 (15.4)	13 (2.4)	161 (29.9)	130 (24.2)
**Responsiveness**					
Diligence of medical examination	16 (3.0)	19 (3.5)	10 (1.9)	294 (54.6)	199 (37.0)
Providing services as pledged	17 (3.2)	14 (2.6)	12 (2.2)	319 (59.3)	176 (32.7)
Following treatment procedures	17 (3.2)	22 (4.1)	7 (1.3)	229 (42.6)	263 (48.9)
**Accessibility**					
I have relaxed admission to the medical professionals I want	21 (3.9)	22 (4.1)	6 (1.1)	243 (45.2)	246 (45.7)
I often receive an arrangement for medical service immediately	20 (3.7)	18 (3.3)	11 (2.0)	274 (50.9)	215 (40.0)
Referral to the higher level of care when need arise	25 (4.6)	26 (4.8)	10 (1.9)	220 (40.9)	257 (47.8)

*Data are presented as the frequency with the corresponding percentage in parentheses*.

#### Dimension of Technical Quality

Less than half (*n* = 256; 47.6%) of postpartum mothers were very satisfied that the hospital gets all it needed to provide thorough medical service; *n* = 252 (46.8%) were satisfied with the competence of health workers; *n* = 295 (54.8%) were satisfied with the professional appearance of healthcare professionals; and *n* = 266 (49.4%) were very satisfied with the good hygienic condition at the hospital (Table 2).

#### Dimension of Communication

Below half **(**256; 47.6%) of postpartum mothers were satisfied to obtain an explanation of diagnosis, treatment, and the reason for medical tests; *n* = 286 (53.2%) were satisfied with the readiness of the healthcare professional to attend to the client; and *n* = 280 (52.0%) were very satisfied for receiving care affectionately and courteously (Table 2).

#### Dimension of Financial

Less than half (*n* = 180; 33.5%) of postpartum mothers were dissatisfied with paying for bills more than they could afford; *n* = 221 (41.1%) were satisfied that they received the medical service they wanted without a financial setback; *n* = 161 (29.9%) were satisfied that items purchase for medical care and use were expensive (Table 2).

#### Dimension of Responsiveness

Most (*n* = 294; 54.6%) of postpartum mothers were satisfied with the diligence of medical examination; *n* = 319 (59.3%) were satisfied with the healthcare professionals providing services as pledged; and *n* = 263 (48.9%) were very satisfied for health workers following treatment procedures (Table 2).

#### Dimension of Accessibility

Below half (*n* = 246; 45.7%) of postpartum mothers were very satisfied with having easy access to the medical specialists they need; *n* = 274 (50.9%) were satisfied with often getting an arrangement for medical service immediately; and *n* = 257 (47.8%) were very satisfied with referral to the higher level of care when need arise (Table 2).

### Levels of Satisfaction of Postpartum Mothers With Skilled Delivery

**Figure 2:**
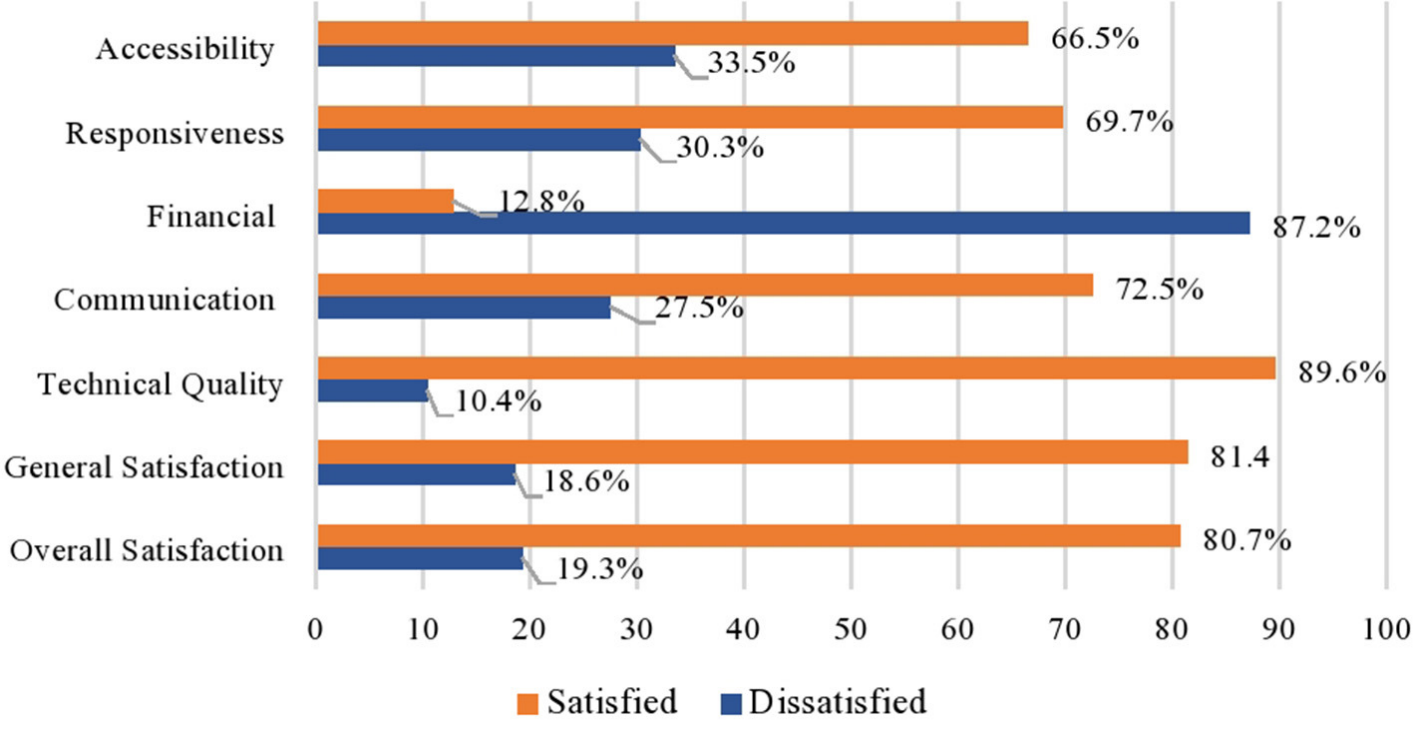
Levels of satisfaction of postnatal mothers with skilled delivery.

The overall satisfaction of postpartum mothers was 434 (80.7%). Regarding the individual dimension of satisfaction, the highest level of satisfaction (*n* = 482; 89.6%) was observed in the technical quality dimension. This is followed by *n* = 438 (81.4%), *n* = 390 (72.5%), *n* = 375 (69.7%), and *n* = 358 (66.5%) in the dimensions of general satisfaction, communication, responsiveness, and accessibility, respectively. The lowest level of satisfaction (*n* = 469; 12.8%) was observed in the financial dimension (Figure 2).

### Factors Associated With Satisfaction of Postpartum Mothers With Skilled Delivery

The independent association of demographics, pregnancy-related factors, and health status before and after childbirth with the various dimensions of skilled delivery and overall satisfaction was determined. The study found no association in the dimensions of overall satisfaction, general satisfaction, technical quality, finance, and accessibility with the study variables (*p* > 0.05).

#### Satisfaction With Communication

The study found odds favoring satisfaction among postpartum mothers aged 25–40 years [crude odds ratio (COR) = 2.31; (95% CI 1.22–4.36), *p* = 0.010] and postpartum mothers who delivered at private healthcare facilities [COR = 1.70; (95% CI 1.00–2.90), *p* = 0.049] compared with their counterparts. Likewise, postpartum mothers who had preterm pregnancy before delivery [COR = 2.08; (95% CI 1.02–4.21), *p* = 0.043] and postpartum mothers who had cesarean section [COR = 2.73; (95% CI 1.05–7.12), *p* = 0.040] were more satisfied likened to their colleagues. Similarly, postpartum mothers who rated the delivery procedures as fair [COR = 2.12; (95% CI 1.15–3.92), *p* = 0.016] and postpartum mothers who presented with complications after childbirth [COR = 2.63; (95% CI 1.09–6.35), *p* = 0.032] were more likely to be satisfied than their counterparts (Table 3).

**Table 3:** Factors associated with satisfaction of skilled delivery in the dimension of communication and responsiveness (*n* = 538).

**Variable**	**Satisfied *n* (%)**	**Dissatisfied *n* (%)**	**Bivariate analysis**		**Multivariate analysis**	
			**COR (95% CI)**	***p*-value**	**AOR (95% CI)**	***p*-value**
**Communication**						
Age (years)						
<25 years	45 (11.5)	30 (20.3)	1		1	
25–40 years	255 (65.4)	92 (62.2)	2.31 (1.22–4.36)	0.010^*^	0.79 (0.13–4.62)	0.792
>40 years	90 (23.1)	26 (17.6)	1.25 (0.76–2.05)	0.381	0.26 (0.01–6.23)	0.405
Types of facility						
Private	82 (21.0)	20 (13.5)	1.70 (1.00–2.90)	0.049^*^	2.38 (0.70–8.08)	0.165
Government	308 (79.0)	128 (86.5)	1		1	
Pregnancy status						
Preterm	51 (13.1)	10 (6.8)	2.08 (1.02–4.21)	0.043^*^	1.60 (0.48–5.38)	0.443
Full-term	339 (86.9)	138 (93.2)	1		1	
Mode of delivery						
Cesarean section	34 (8.7)	5 (3.4)	2.73 (1.05–7.12)	0.040^*^	1.36 (0.13–14.17)	0.797
Spontaneous vagina delivery	356 (91.3)	143 (96.6)	1		1	
Delivery procedure						
Poor	49 (12.6)	32 (21.6)	1		1	
Fair	250 (64.1)	88 (59.5)	2.12 (1.15–3.92)	0.016^*^	0.66 (0.12–3.72)	0.639
Good	91 (23.3)	28 (18.9)	1.14 (0.70–1.86)	0.589	4.39 (0.23–85.22)	0.329
Presence of complication						
Yes	39 (10.0)	6 (4.1)	2.63 (1.09–6.35)	0.032^*^	1.01 (0.08–12.82)	0.991
No	351 (90.0)	142 (95.9)	1		1	
**Responsiveness**						
Age (years)						
<25 years	59 (15.7)	16 (9.8)	1		1	
25–40 years	245 (65.3)	102 (62.6)	1.54 (0.84–2.79)	0.161	0.74 (0.08–6.77)	0.788
>40 years	71 (18.9)	45 (27.6)	2.34 (1.20–4.55)	0.013^*^	0.23 (0.01–6.27)	0.386
Parity						
Primipara	71 (18.9)	45 (27.6)	1			
Multipara	304 (81.1)	118 (72.4)	1.63 (1.06–2.51)	0.025^*^	–	
Delivery procedure						
Poor	64 (17.1)	17 (10.4)	1		1	
Fair	239 (63.7)	99 (60.7)	1.56 (0.87–2.80)	0.136	2.07 (0.24–17.96)	0.509
Good	72 (19.2)	47 (28.8)	2.46 (1.28–4.70)	0.007^**^	10.00 (0.40–250.42)	0.161

*Significant at*

* *p < 0.05*,

** *p < 0.01; CI, confidence interval; COR, crude odds ratio; AOR, adjusted odds ratio; 1 is the reference*.

#### Satisfaction With Responsiveness

The study also found an association between age, parity and delivery procedure, and the responsiveness dimension of skilled delivery. The odds ratio revealed that postpartum mothers aged above 40 years were 2.34 times more likely to be satisfied than those aged <25 years [COR = 2.34; (95% CI 1.20–4.55), *p* = 0.013]. Postpartum mothers who were multipara were 1.63 times more likely to be satisfied than primipara mothers [COR = 1.63; (95% CI 1.06–2.51), *p* = 0.025]. The postpartum mothers who rated their delivery procedures as good were 2.46 times more likely to be satisfied than those who rated them as poor [adjusted odds ratio (aOR) = 2.19; (95% CI 1.28–4.70), *p* = 0.007] (Table 3).

### Correlation Between the Satisfaction of Postpartum Mothers and Study Variables

This study found no correlation between the overall satisfaction of postpartum mothers and the study variables. However, overall satisfaction was significantly and positively correlated with the various dimensions of skilled delivery (Table 4).

**Table 4:** Pearson correlation coefficient of satisfaction of postpartum mothers with study variables.

**Variable**	**1**	**2**	**3**	**4**	**5**	**6**	**7**	**8**	**9**	**10**	**11**	**12**	**13**	**14**	**15**	**16**
1	1	−0.03	−0.82^**^	−0.07	−0.76^**^	−0.37^**^	−0.44^**^	0.98^**^	−0.47^**^	−0.01	0.00	−0.03	−0.11^*^	−0.03	0.11^**^	0.06
2	−0.03	1	0.05	0.03	0.06	−0.05	0.03	−0.04	0.02	0.08	0.03	0.08	0.09^*^	−0.01	0.09^*^	0.07^*^
3	−0.07	0.03	0.09^*^	1	0.09^*^	0.03	0.01	−0.07	0.01	−0.05	0.01	0.04	−0.06	−0.03	−0.01	−0.08
4	−0.76^**^	0.06	0.92^**^	0.09^*^	1	0.50^**^	0.58^**^	−0.74^**^	0.63^**^	0.05	0.01	0.06	0.09^*^	−0.00	−0.05	−0.04
5	−0.82^**^	0.05	1	0.09^*^	0.92^**^	0.47^**^	0.53^**^	−0.81^**^	0.58^**^	0.02	−0.01	0.05	0.06	−0.01	−0.10^*^	−0.07
6	−0.37^**^	−0.05	0.47^**^	0.03	0.50^**^	1	0.78^**^	−0.37^**^	0.85^**^	0.07	0.04	0.05	0.09^*^	0.06	−0.02	−0.02
7	−0.44^**^	0.03	0.53^**^	0.01	0.58^**^	0.78^**^	1	−0.43^**^	0.93^**^	0.06	0.02	0.07	0.09^*^	0.04	−0.03	−0.01
8	0.98^**^	−0.04	−0.81^**^	−0.07	−0.74^**^	−0.37^**^	−0.43^**^	1	−0.46^**^	0.01	0.00	−0.03	−0.10^*^	−0.02	0.12^**^	0.07
9	−0.47^**^	0.02	0.58^**^	0.01	0.63^**^	0.85^**^	0.93^**^	−0.46^**^	1	0.08	0.02	0.08	0.10^*^	0.07	−0.02	−0.01
10	−0.01	0.08	0.02	−0.05	0.05	0.07	0.06	0.01	0.08	1	0.57^**^	0.57^**^	0.63^**^	0.19^**^	0.61^**^	0.50^**^
11	0.00	0.03	−0.01	0.01	0.01	0.04	0.02	0.00	0.02	0.57^**^	1	0.28^**^	0.41^**^	0.11^**^	0.31^**^	0.22^**^
12	−0.03	0.08	0.05	0.04	006	0.05	0.07	−0.03	0.08	0.57^**^	0.28^**^	1	0.40^**^	0.09^*^	0.40^**^	0.40^**^
13	−0.11^*^	0.09^*^	0.06	−0.06	0.09^*^	0.09^*^	0.09^*^	−0.10^*^	0.10^*^	0.63^**^	0.41^**^	0.40^**^	1	0.10^*^	0.36^**^	0.29^**^
14	−0.03	−0.01	−0.01	−0.03	−0.00	0.06	0.04	−0.02	0.07	0.19^**^	0.11^**^	0.09^*^	0.10^*^	1	0.12^**^	0.05
15	0.11^**^	0.09^*^	−0.10^*^	−0.01	−0.05	−0.02	−0.03	0.12^**^	−0.02	0.61^**^	0.31^**^	0.40^**^	0.36^**^	0.12^**^	1	0.42^**^
16	0.06	0.09^*^	−0.07	−0.08	−0.04	−0.02	0–0.01	0.07	−0.01	0.50^**^	0.22^**^	0.40^**^	0.29^**^	0.05	0.42^**^	1

*1, Age; 2, Educational status; 3, Service payment; 4, Type of facility; 5, Parity; 6, Pregnancy status; 7, Mode of delivery; 8, Delivery procedure; 9, Presence of complication; 10, Overall satisfaction; 11, General satisfaction; 12, Technical quality; 13, Communication; 14, Financial; 15, Responsiveness; 16, Accessibility; Significant at*

* *p < 0.05*,

** *p < 0.01*.

## Discussion

The present study assessed the association between pregnancy-related factors and health status before and after childbirth with satisfaction with skilled delivery in multiple dimensions among postpartum mothers in the Akatsi South District, Ghana. This study showed that overall more than half of the postpartum mothers were satisfied with the skilled deliveries. This would help to increase the utilization of services and treatment compliance and promote good relations and evaluation of healthcare providers based on the perspective of the mothers. Our finding contradicts earlier studies conducted in Ethiopia and Nigeria (3, 8) but agrees with studies performed in Turkey, Ethiopia, Ghana, and China (5, 9, 14, 25).

The disproportion across the studies could be the sample size, the cut-off point used, the study setting, and the subjectivity of the postpartum mothers in determining their satisfaction. Another justification for these disparities in the results could be that the quality of services rendered in various countries and within countries from one setting to another setting might inform the measurement of satisfaction using different methods. Similar studies conducted in Nepal, Nigeria, the Netherlands, Ghana, and Australia assessed patient satisfaction in several dimensions, but the overall satisfaction scores were not calculated (7, 10–12, 21). The discrepancy could be due to the availability of services rendered in our study setting compared to those in previous studies.

This study found good satisfaction in five dimensions of skilled delivery out of the six dimensions assessed and overall satisfaction. This implies that postpartum mothers have confidence and trust in the services being provided, and they are likely to continue assessing healthcare services from these health facilities or may not be because of their dissatisfaction in the financial dimension. These findings collaborate with studies conducted in Ethiopia, Nigeria, Nepal, and Australia (7, 10, 11, 14) but oppose studies done in Ghana and the Netherlands (5, 21). These differential satisfaction scores could be due to the unavailability of anticipated services, inadequate healthcare facilities, and lack of healthcare providers in these study settings.

It could also be due to the variances in healthcare locations, funding systems, and the precedence specified to mother satisfaction by regulatory bodies. Not surprisingly, our study reported dissatisfaction with the financial dimension of skilled delivery, which contradicts studies done in Nepal and Australia (7, 10). The probable reason could be that most of the postpartum mothers in our study were dissatisfied with paying for bills more than they could afford, although the government of Ghana instigated an NHIS to offer free healthcare to all citizens.

In our study, the OR showed that postpartum mothers aged between 25 and 40 years and above 40 years were more likely to be satisfied with skilled delivery than those aged below 25 years in the dimensions of communication and responsiveness, respectively. Our findings confirm studies conducted in Nepal and Australia (7, 32) but differ from studies performed in Ethiopia and Turkey (8, 9). These disparities could be due to many factors. First, the possible reason could be the metamorphoses in the treatments provided of which the older population is likely to accept based on their experiences than the younger population (7). Second, the older populace is generally more relaxed with the authoritarian type of care rather than patient-centered care (7). Additionally, the older population was more sociable, respectful, had low expectations, and tolerant toward healthcare providers than the younger population (8).

Our study showed that postpartum mothers who were multiparous were more likely to be satisfied with skilled delivery than those who were primiparous, particularly, in the dimension of responsiveness. Similar findings were found in studies conducted in Nepal, the United States of America, and the Netherlands (1, 21, 33). In contrast to our study, researches conducted in Lebanon and Australia (34, 35) found that primiparous women were more satisfied with maternal services. The possible reason could be that the multiparous mothers had experience and had a better understanding of the challenges of skilled delivery, which confidently impacted their delivery skills. Another probable reason could be that multiparous mothers have fewer expectations from healthcare providers, which influence their decision-making during labor.

This study found that postpartum mothers who delivered at private healthcare facilities were more likely to be satisfied with skilled delivery than those who delivered at government facilities, explicitly in the dimension of communication. This finding opposes a study done in Nepal (1). These variations might be due to several reasons. First, it could be due to the health insurance services handled in these study settings, although in Ghana, it is not restricted directly either to the government or private healthcare facility, which means that citizens are required to use the services by registration. Second, private facilities might not have enough money to subsidize the medical cost and other services available, but the communication and the relationship between the patients and healthcare providers might increase their satisfaction. Another probable reason could be that private health facilities might have many services required for skilled delivery that are unavailable in government facilities.

Our study showed that postpartum mothers who had preterm pregnancies before delivery were more likely to be satisfied than those who had a full-term pregnancy, specifically with the dimension of communication of skilled delivery. This could be attributed to many factors. The likely justification could be that postpartum mothers with preterm pregnancies are aware of having premature babies that mostly end up with major illnesses. Another probable reason could be that postpartum mothers with preterm pregnancies are not worried about staying longer in the healthcare facility because their babies need to be monitored for neonatal danger signs before being discharged home. Our findings indicate the impact of pregnancy status on the satisfaction of postpartum mothers with skilled delivery, although no studies were found to compare the findings. Henceforth, another study is recommended to verify our findings.

This study revealed that postpartum mothers who had cesarean section were more likely to be satisfied with skilled delivery in the dimension of communication compared to mothers who had a spontaneous vaginal delivery. This finding disagreed with studies conducted in the Netherlands, Israel, Ethiopia, and China (21, 24, 36, 37) but was consistent with a study performed in Nepal (1). The likely clarification could be that undergoing a cesarean section of delivery is frequently linked with an undesirable labor experience. Another probable reason could be that the cesarean section of delivery is most often associated with complications that are expected to affect the satisfaction of skilled delivery because it requires wide collaboration.

Our study showed that postpartum mothers who rated their delivery procedures as fair and good were more likely to be satisfied mainly in the dimensions of communication and responsiveness, respectively, compared to those who rated theirs as poor. This finding is consistent with a study conducted in the Netherlands (21). These two studies emphasize that averting abuse and reaching optimum client-care provider communication and receptiveness during labor and childbirth are greatly significant. Courtesy of respectful skilled delivery is required to certify an optimistic experience for all women. Hence, dutiful service delivery must not only demand the avoidance of maltreatment during care.

Our study revealed that postpartum mothers who had complications after childbirth were more likely to be satisfied in the dimension of communication than mothers who had no complications after childbirth. Our finding is incongruent with studies conducted in Ethiopia (2, 24). In studies performed in Nepal, Ghana, and the Netherlands (1, 4, 21), no associations were found between complications after childbirth and the satisfaction of skilled delivery among postpartum mothers. The probable reason is that complications after childbirth require medical interventions and require more widespread and demanding interactions between postpartum mothers and care providers, which might affect their satisfaction with skilled delivery.

This study found no correlation between the overall satisfaction of skilled delivery and study variables, which contradicts a study conducted in Ghana (5). The possible reason could be due to the dissimilarity of the study population, study setting, and scoring point used. In our study, the overall satisfaction of postpartum mothers was significantly and positively correlated with the various dimensions of skilled delivery. Unfortunately, no studies have found assessing the correlation between the overall satisfaction of postpartum mothers with the dimensions of skilled delivery, and this generates problems of comparing our findings to provide appropriate justification. Although these dimensions of skilled delivery are firmly liberated from each other, they indicate the importance of providing equal services irrespective of the dimensions to postpartum mothers by healthcare professionals.

## Strength and Limitation

Our study was the first to assess the association between pregnancy-related factors and health status before and after childbirth with satisfaction with skilled delivery in multiple dimensions among postpartum mothers in the Akatsi South District, Ghana. Furthermore, because of the descriptive landscape of the sample used, the results of our study could be widespread to primary health facilities in the district and beyond. Our findings offer a pragmatic signal for Ghana Health Services and the Ministry of Health to encourage their representative health agencies to continue to provide patient-centered care to increase satisfaction at every healthcare facility. This study also provides empirical findings to the Health Directorate in Akatsi South District to evaluate the performance of their staff and award their outstanding facility.

Although our study added different standpoints in the zone of satisfaction of postpartum mothers in Ghana, it has limitations. First, in deducing our findings, it is significant to be aware that statistically significant disparities do not automatically signpost clinical application. Our findings should not be widespread above hospitals at the district level due to the disparities in the healthcare services provided. Third, the existence of a social desirability bias might equally be added to the difference within the interview-answered questionnaires. Other factors could have added to the satisfaction scores of postpartum mothers, which our study could not assess due to the unavailability of literature in the study setting. Additionally, the unattainability of appropriate findings from related studies in the district and the region hinders the comparison of our findings to define precise interventions; hence, our findings should be deduced and interpreted with cautiousness.

## Conclusion

Overall, more than three-quarters of the postpartum mothers were satisfied with the skilled delivery. The dimension with the highest satisfaction level was technical quality, and the dimension with the lowest satisfaction was the financial dimension. Age, type of facility, parity, pregnancy status, mode of delivery, delivery procedure, and the presence of complications were significantly associated with satisfaction with skilled delivery. The Ghana Health Service should use these findings cautiously to deliver a policy for the continued improvement of skilled delivery. The Ministry of Health should conduct comparable studies frequently in different districts across the country to apprehend a broader illustration of the satisfaction of postpartum mothers with skilled delivery. The district health directorate should provide interventions that are targeted directly to mend the various dimensions of the satisfaction of postnatal mothers with skilled delivery with low scores. Further studies should assess other pregnancy-related factors and health statuses before and after childbirth to obtain innovative discernments on satisfaction from a standardized standpoint.

## Data Availability Statement

The raw data supporting the conclusions of this article will be made available by the authors, without undue reservation.

## Ethics Statement

The studies involving human participants were reviewed and approved by Research Ethics Committee (REC) of the University of Health and Allied Sciences (UHAS), Ho. Protocol identification number (UHAS-REC No: A.5 [78] 17-18) was granted. Written informed consent to participate in this study was provided by the participants' legal guardian/next of kin.

## Author Contributions

LT and CA conceptualized and designed the study. LT drafted and wrote the manuscript. LT, CSB, VK, JT, LA, MA, CB, and SS coordinated and participated in the data collection. LT, CA, and QL contributed to the data analysis, interpretation, and discussion. All authors reviewed the manuscript for intellectual content, and each author approved the final manuscript.

## Conflict of Interest

The authors declare that the research was conducted in the absence of any commercial or financial relationships that could be construed as a potential conflict of interest.

## Publisher's Note

All claims expressed in this article are solely those of the authors and do not necessarily represent those of their affiliated organizations, or those of the publisher, the editors and the reviewers. Any product that may be evaluated in this article, or claim that may be made by its manufacturer, is not guaranteed or endorsed by the publisher.

## Abbreviations

WHO: World Health Organization
PNC: Postnatal Care
ANC: Antenatal Care
IBM SPSS: Statistical Package for Social Sciences
REC: Research Ethics Committee
UHAS: University of Health and Allied Sciences
NHIS: National Health Insurance Scheme
AOR: Adjusted Odds Ratio
COR: Crude Odds Ratio
Cl: Confidence Interval
PSQ: Patient Satisfaction Questionnaire
PSNCQQ: Patient Satisfaction with Nursing Care Quality Questionnaire
SQPS: Self-reporting Questionnaire on Patient Satisfaction
NSNS: Newcastle Satisfaction with Nursing Scale.

## References

[1] Panth A Kafle P. Maternal satisfaction on delivery service among postnatal mothers in a Government Hospital, Mid-Western Nepal. Obstet Gynecol Int. (2018) 1:1–12. 10.1155/2018/4530161 30034472 PMC6035828

[2] Negero MG Mitike YB Worku AG Abota TL. Skilled delivery service utilization and its association with the establishment of Women's Health Development Army in Yeky district, South West Ethiopia: a multilevel analysis. BMC Res Notes. (2018) 11:83. 10.1186/s13104-018-3140-0 29382372 PMC5791222

[3] Obi IE. Patient satisfaction with services at a tertiary hospital in south-east Nigeria. Malawi Med J. (2018) 30:270–5. 10.4314/mmj.v30i4.10 31798806 PMC6863408

[4] Adjei KK Kikuchi K Owusu-Agyei S Enuameh Y Shibanuma A Ansah EK. Women's overall satisfaction with health facility delivery services in Ghana: a mixed-methods study. Trop Med Health. (2019) 47:1–9. 10.1186/s41182-019-0172-7 31320830 PMC6612170

[5] Odonkor ST Frimpong C Duncan E Odonkor C. Trends in patients' overall satisfaction with healthcare delivery in Accra, Ghana. African J Prim Heal Care Fam Med. (2019) 11:1–6. 10.4102/phcfm.v11i1.1884 31588770 PMC6779968

[6] Bai G Korfage IJ Mautner E Raat H. Determinants of maternal health-related quality of life after childbirth: the generation R study. Int J Environ Res Public Health. (2019) 16:1–12. 10.3390/ijerph16183231 31487782 PMC6765914

[7] Adhikari M Paudel NR Mishra SR Shrestha A Upadhyaya DP. Patient satisfaction and its socio-demographic correlates in a tertiary public hospital in Nepal: a cross-sectional study. BMC Health Serv Res. (2021) 21:135. 10.1186/s12913-021-06155-3 33579283 PMC7881603

[8] Ayele Y Hawulte B Feto T Basker GV Bacha YD. Assessment of patient satisfaction with pharmacy service and associated factors in public hospitals, Eastern Ethiopia. SAGE Open Med. (2020) 8:1–7. 10.1177/2050312120922659 32435492 PMC7223202

[9] Karaca A Durna Z. Patient satisfaction with the quality of nursing care. Nurs Open. (2019) 6:535–45. 10.1002/nop2.237 30918704 PMC6419107

[10] Ifediora CO Rogers GD. Levels and predictors of patient satisfaction with doctor home-visit services in Australia. Fam Pract. (2017) 34:63–70. 10.1093/fampra/cmw092 27587567

[11] Umoke M Umoke PCI Nwimo IO Nwalieji CA Onwe RN Emmanuel Ifeanyi N. Patients' satisfaction with quality of care in general hospitals in Ebonyi State, Nigeria, using SERVQUAL theory. SAGE open Med. (2020) 8:1–9. 10.1177/2050312120945129 32782795 PMC7385818

[12] Aninanya GA Otupiri E Howard N. Effects of combined decision-support and performance-based incentives on reported client satisfaction with maternal health services in primary facilities: a quasi-experimental study in the Upper East Region of Ghana. PLoS ONE. (2021) 16:e0249778. 10.1371/journal.pone.0249778 33878127 PMC8057590

[13] Berger S Saut AM Berssaneti FT. Using patient feedback to drive quality improvement in hospitals: a qualitative study. BMJ Open. (2020) 1:e037641. 10.1136/bmjopen-2020-037641 33099495 PMC7590344

[14] Geberu DM Biks GA Gebremedhin T Mekonnen TH. Factors of patient satisfaction in adult outpatient departments of private wing and regular services in public hospitals of Addis Ababa, Ethiopia: a comparative cross-sectional study. BMC Health Serv Res. (2019) 19:869. 10.1186/s12913-019-4685-x 31752821 PMC6873435

[15] Atinga RA Abekah-Nkrumah G Domfeh KA. Managing healthcare quality in Ghana: a necessity of patient satisfaction. Int J Health Care Qual Assur. (2011) 24:548–63. 10.1108/09526861111160580 22204088

[16] Kwateng KO Lumor R Acheampong FO. Service quality in public and private hospitals: a comparative study on patient satisfaction. Int J Healthc Manag. (2017) 1:1–9. 10.1080/20479700.2017.1390183

[17] Abekah-Nkrumah G Nkrumah J. Perceived work environment and patient-centered behavior: a study of selected district hospitals in the central region of Ghana. PLoS ONE. (2021) 16:e0244726. 10.1371/journal.pone.0244726 33493181 PMC7833094

[18] Wudu MA. Predictors of adult patient satisfaction with inpatient nursing care in Public Hospitals of Eastern Amhara Region, Northeastern Ethiopia, 2020. Patient Prefer Adher. (2021) 15:177–85. 10.2147/PPA.S294041 33564228 PMC7867496

[19] Desta H Berhe T Hintsa S. Assessment of patients' satisfaction and associated factors among outpatients received mental health services at public hospitals of Mekelle Town, northern Ethiopia. Int J Ment Health Syst. (2018) 12:1–7. 10.1186/s13033-018-0217-z 30008801 PMC6042256

[20] Olamuyiwa TE Adeniji FO. Patient's Satisfaction With Quality of Care at a National Health Insurance Clinic at a Tertiary Center, South-South Nigeria. J Patient Exp. (2021) 8:1–7. 10.1177/2374373520981471 34179352 PMC8205399

[21] van der Pijl MSG Kasperink M Hollander MH Verhoeven C Kingma E de Jonge A. Client-care provider interaction during labour and birth as experienced by women: respect, communication, confidentiality and autonomy. PLoS ONE. (2021) 16:e0246697. 10.1371/journal.pone.0246697 33577594 PMC7880498

[22] Pettersson ML Nedstrand E Bladh M Svanberg AS Lampic C Sydsjö G. Mothers who have given birth at an advanced age-health status before and after childbirth. Sci Rep. (2020) 10:1–15. 10.1038/s41598-020-66774-4 32546715 PMC7298035

[23] Kebede H Tsehay T Necho M Zenebe Y. Patient satisfaction towards outpatient pharmacy services and associated factors at Dessie Town Public Hospitals, South Wollo, North-East Ethiopia. Patient Prefer Adher. (2021) 15:87–97. 10.2147/PPA.S287948 33519194 PMC7837535

[24] Tayelgn A Zegeye DT Kebede Y. Mothers' satisfaction with referral hospital delivery service in Amhara Region, Ethiopia. BMC Pregn Childb. (2011) 11:78. 10.1186/1471-2393-11-78 22023913 PMC3254067

[25] Liu L Fang J. Study on potential factors of patient satisfaction: based on exploratory factor analysis. Patient Prefer Adher. (2019) 13:1983–94. 10.2147/PPA.S228073 31819380 PMC6885779

[26] Amporfro DA Boah M Yingqi S Wabo TMC Zhao M Nkondjock VRN. Patients satisfaction with healthcare delivery in Ghana. BMC Health Serv Res. (2021) 21:722. 10.1186/s12913-021-06717-5 34294102 PMC8299658

[27] Ghana Health Service GHS. Annual Health Report, Akatsi South District (2020).

[28] Ghana Statistical Service GSS. Population and Housing Census 2010 District and Analytical Report (2010).

[29] Tuglo LS Agordoh PD Tekpor D Pan Z Agbanyo G Chu M. Food safety knowledge, attitude, and hygiene practices of street-cooked food handlers in North Dayi District, Ghana. Environ Health Prev Med. (2021) 26:1–13. 10.1186/s12199-021-00975-9 33941082 PMC8091506

[30] Monica 1776 Main Street Santa, California 90401 – 3208. Patient Satisfaction Questionnaires (PSQ-III and PSQ-18). Avaialble online at: https://www.rand.org/health-care/%0Asurveys_tools/psq.html (accessed March 10, 2018).

[31] Tuglo LS Nyande FK Agordoh PD Nartey EB Pan Z Logosu L. Knowledge and practice of diabetic foot care and the prevalence of diabetic foot ulcers among diabetic patients of selected hospitals in the Volta Region, Ghana. Int Wound J. (2021) 18:1–14. 10.1111/iwj.13656 34190402 PMC8874051

[32] Hale A Coombes I Stokes J Aitken S Clark F Nissen L. Patient satisfaction from two studies of collaborative doctor-pharmacist prescribing in Australia. Health Expect. (2016) 19:49–61. 10.1111/hex.12329 25614342 PMC5055216

[33] Neerland CE Avery MD Saftner MA Gurvich OV. Maternal confidence for physiologic birth: associated prenatal characteristics and outcomes. Midwifery. (2019) 77:110–6. 10.1016/j.midw.2019.07.004 31319365

[34] Al Ahmar E Tarraf S. Assessment of the socio-demographic factors associated with the satisfaction related to the childbirth experience. Open J Obstet Gynecol. (2014) 4:1–28. 10.4236/ojog.2014.410083

[35] Jenkins MG Ford JB Morris JM Roberts CL. Women's expectations and experiences of maternity care in NSW–what women highlight as most important. Women and Birth. (2014) 27:214–9. 10.1016/j.wombi.2014.03.002 24746379

[36] Handelzalts JE Waldman Peyser A Krissi H Levy S Wiznitzer A Peled Y. Indications for emergency intervention, mode of delivery, and the childbirth experience. PLoS ONE. (2017) 12:e0169132. 10.1371/journal.pone.0169132 28046019 PMC5207782

[37] Yang X-J Sun S-S. Comparison of maternal and fetal complications in elective and emergency cesarean section: a systematic review and meta-analysis. Arch Gynecol Obstet. (2017) 296:503–12. 10.1007/s00404-017-4445-2 28681107

