# Corrigendum: The association between pregnancy-related factors and health status before and after childbirth with satisfaction with skilled delivery in multiple dimensions among postpartum mothers in the Akatsi South District, Ghana

**DOI:** 10.3389/fpubh.2022.1010907

**Published:** 2022-09-14

**Authors:** Lawrence Sena Tuglo, Comfort Agbadja, Cynthia Sekyere Bruku, Vivian Kumordzi, Jessica Dzigbordi Tuglo, Leticia Atiah Asaaba, Mercy Agyei, Cynthia Boakye, Sylvia Mawusinu Sakre, Qingyun Lu

**Affiliations:** ^1^Department of Epidemiology, School of Public Health, Nantong University, Nantong, China; ^2^Department of Nutrition and Dietetics, School of Allied Health Sciences, University of Health and Allied Sciences, Ho, Ghana; ^3^Diettherapy Department, Ho Teaching Hospital, Ho, Ghana; ^4^Department of Midwifery, School of Nursing and Midwifery, University of Health and Allied Sciences, Ho, Ghana; ^5^Obstetrics and Gynaecology Department, Saint (ST) Dominic Hospital, Akwatia, Ghana; ^6^Ashaiman Municipal Health Directorate, Ashaiman, Ghana; ^7^Community Health Department, Evangelical Presbyterian Mimi Clinic, Adaklu, Ghana; ^8^Maternity Department, Madina Polyclinic Kekele, Madina, Ghana; ^9^Maternity Department, Ga South Municipal Hospital, Waija, Ghana; ^10^Maternity Department, Eastern Regional Hospital, Koforidua, Ghana; ^11^Department of Community Health Nursing, Nurses Training College, Ho, Ghana

**Keywords:** pregnancy-related factors, health status before and after childbirth, satisfaction, skilled delivery, multiple dimensions, postpartum mothers, Ghana

In the original article, there was an error with **affiliation** 12. **Affiliation** 12 should not appear and Author Qingyun Lu should be affiliated with **affiliation** 1. **Affiliation** and **author list** should appear, respectively, as follows:

^“1^ Department of Epidemiology, School of Public Health, Nantong University, Nantong, China

^2^ Department of Nutrition and Dietetics, School of Allied Health Sciences, University of Health and Allied Sciences, Ho, Ghana

^3^ Diettherapy Department, Ho Teaching Hospital, Ho, Ghana

^4^ Department of Midwifery, School of Nursing and Midwifery, University of Health and Allied Sciences, Ho, Ghana

^5^ Obstetrics and Gynaecology Department, Saint (ST) Dominic Hospital, Akwatia, Ghana

^6^ Ashaiman Municipal Health Directorate, Ashaiman, Ghana

^7^ Community Health Department, Evangelical Presbyterian Mimi Clinic, Adaklu, Ghana

^8^ Maternity Department, Madina Polyclinic Kekele, Madina, Ghana

^9^ Maternity Department, Ga South Municipal Hospital, Waija, Ghana

^10^ Maternity Department, Eastern Regional Hospital, Koforidua, Ghana

^11^ Department of Community Health Nursing, Nurses Training College, Ho, Ghana”

“Lawrence Sena Tuglo^1, 2, 3^, Comfort Agbadja^4^, Cynthia Sekyere Bruku^4, 5^, Vivian Kumordzi^4, 6^, Jessica Dzigbordi Tuglo^4, 7^, Leticia Atiah Asaaba^4, 8^, Mercy Agyei^4, 9^, Cynthia Boakye^4, 10^, Sylvia Mawusinu Sakre^11^ and Qingyun Lu^1^*”

The authors apologize for this error and state that this does not change the scientific conclusions of the article in any way. The original article has been updated.

## Publisher's note

All claims expressed in this article are solely those of the authors and do not necessarily represent those of their affiliated organizations, or those of the publisher, the editors and the reviewers. Any product that may be evaluated in this article, or claim that may be made by its manufacturer, is not guaranteed or endorsed by the publisher.

